# Minimum milk feeding frequency and its associated factors among non-breastfed children aged 6–23 months in sub-saharan Africa: a multilevel analysis of the recent demographic and health survey data

**DOI:** 10.1186/s12889-024-19275-2

**Published:** 2024-06-28

**Authors:** Enyew Getaneh Mekonen, Belayneh Shetie Workneh, Mohammed Seid Ali, Almaz Tefera Gonete, Tewodros Getaneh Alemu, Tadesse Tarik Tamir, Berhan Tekeba, Masresha Asmare Techane, Mulugeta Wassie, Alemneh Tadesse Kassie, Alebachew Ferede Zegeye

**Affiliations:** 1 Department of Surgical Nursing, School of Nursing, College of Medicine and Health Sciences, University of Gondar, Gondar, Ethiopia; 2 Department of Emergency and Critical Care Nursing, School of Nursing, College of Medicine and Health Sciences, University of Gondar, Gondar, Ethiopia; 3 Department of Pediatrics and Child Health Nursing, School of Nursing, College of Medicine and Health Sciences, University of Gondar, Gondar, Ethiopia; 4 School of Nursing, College of Medicine and Health Sciences, University of Gondar, Gondar, Ethiopia; 5 Department of Clinical Midwifery, School of Midwifery, College of Medicine and Health Sciences, University of Gondar, Gondar, Ethiopia; 6 Department of Medical Nursing, School of Nursing, College of Medicine and Health Sciences, University of Gondar, Gondar, Ethiopia

**Keywords:** Minimum milk feeding frequency, Non-breastfed children, DHS, Sub-saharan Africa, Multilevel analysis

## Abstract

**Background:**

Poor infant and child feeding practices, in combination with increased rates of infectious diseases, are the main immediate causes of malnutrition during the first two years of life. Non-breastfed children require milk and other dairy products, as they are rich sources of calcium and other nutrients. As far as our search is concerned, there is no evidence on the pooled magnitude and determinants of minimum milk feeding frequency among non-breastfed children in sub-Saharan Africa conducted using the most recent indicators for assessing infant and young child feeding practices published in 2021. Therefore, this study is intended to determine the magnitude and associated factors of minimum milk feeding frequency among non-breastfed children aged 6–23 months in sub-Saharan Africa using the most recent guideline and demographic and health survey dataset.

**Methods:**

Data from the most recent health and demographic surveys, which were carried out between 2015 and 2022 in 20 sub-Saharan African countries, were used. The study comprised a weighted sample consisting of 13,315 non-breastfed children between the ages of 6 and 23 months. STATA/SE version 14.0 statistical software was used to clean, recode, and analyze data that had been taken from DHS data sets. Utilizing multilevel mixed-effects logistic regression, the factors associated with the outcome variable were identified. Model comparison and fitness were assessed using deviance (-2LLR), likelihood ratio test, median odds ratio, and intra-class correlation coefficient. Finally, variables with a p-value < 0.05 and an adjusted odds ratio with a 95% confidence interval were declared statistically significant.

**Results:**

The pooled magnitude of minimum milk feeding frequency among non-breastfed children aged 6–23 months in sub-Saharan African countries was 12.39% (95% CI: 11.85%, 12.97%). Factors like maternal educational level [AOR = 1.61; 95% CI (1.35, 1.91)], marital status of the mother [AOR = 0.77; 95% CI (0.67, 0.89)], maternal working status [AOR = 0.80; 95% CI (0.71, 0.91)], media exposure [AOR = 1.50; 95% CI (1.27, 1.77)], wealth index [AOR = 1.21; 95% CI (1.03, 1.42)], place of delivery [AOR = 1.45; 95% CI (1.22, 1.72)], ANC visit attended during pregnancy [AOR = 0.49; 95% CI (0.39, 0.62)], PNC checkup [AOR = 1.57; 95% CI (1.40, 1.76)], child’s age [AOR = 0.70; 95% CI (0.53, 0.93)], and residence [AOR = 2.15; 95% CI (1.87, 2.46)] were significantly associated with minimum milk feeding frequency.

**Conclusions:**

In sub-Saharan Africa, the proportion of minimum milk feeding frequency among non-breastfed children aged between 6 and 23 months was low. The likelihood of minimum milk feeding frequency increases with high levels of education, unemployment, media exposure, rich wealth status, being unmarried, having a child born in a health facility, getting PNC checks, being between 6 and 8 months old, and living in an urban area. Hence, promoting women’s education, increasing the economic status of the household, disseminating nutrition information through media, strengthening maternal health service utilization like health facility delivery and PNC services, and giving prior attention to mothers with older children and from rural areas are strongly recommended.

**Supplementary Information:**

The online version contains supplementary material available at 10.1186/s12889-024-19275-2.

## Background

During infancy and early childhood, sufficient nutrition is vital to the improvement of each child’s complete human power [1]. The period from birth to 24 months of age is fundamental to the advancement of ideal growth, well-being, and behavioral development and is the critical age for growth retardation, micronutrient deficiencies, and public childhood diseases like diarrhea [2]. Reversing child undernutrition, especially stunting that has occurred earlier, is very difficult after a child reaches two years of age [3]. Inadequate nutrition during this critical window has both immediate and long-term consequences. The immediate consequences of early nutritional insufficiencies include substantial morbidity and mortality, as well as delayed mental and motor development. Deficiencies in academic performance, work capability, reproductive consequences, and overall well-being during the teenage years and middle age are the long-term consequences [4]. The cycle of malnutrition is continuous, as the probability of giving birth to an undernourished, low-birth-weight infant is higher when a malnourished girl child grows up [5]. Poor infant and child feeding practices, in combination with increased rates of infectious diseases, are the main immediate causes of malnutrition during the first two years of life [6].

The World Health Organization’s (WHO) Global Strategy for Infant and Young Child Feeding recommends that children continue breastfeeding for 24 months or beyond [7]. Breastfeeding for a long period of time decreases the odds of chronic illnesses in babyhood, like pneumonia and diarrhea, enhances the immune system of the child, and helps in the appropriate development of the brain [8]. But there are a number of children who will not be able to obtain the benefits of breastfeeding in the early months of life or for whom breastfeeding will discontinue before the recommended duration [9]. Rapid early termination of breastfeeding carries threats for the infant: dehydration, refusal to eat, loss of bonding with the mother, loss of sucking (comfort), psychological trauma, increased risk of neglect, malnutrition, illness, or death [10]. Only half of children aged 6 to 23 months are fed the minimum number of meals a day for their age [11]. Only one in every six children is receiving a minimum acceptable diet globally [12]. Non-breastfed infants and young children need at least 400–600 mL/d of extra fluids (in addition to the 200–700 mL/d of water that is estimated to come from milk and other foods) in a temperate climate and 800–1200 mL/d in a hot climate, as breast milk is almost 90% water [13].

Non-breastfed children require milk and other dairy products as they are rich sources of calcium and other nutrients [14]. According to the WHO guiding principles for feeding non-breastfed children aged 6–23 months, the amount of milk needed to achieve nutrient requirements depends on the other foods consumed by the child [9]. When the child’s diet does not include fortified foods or supplements, the daily requirement of milk is about 200–400 mL if other animal-source foods are included in the diet and about 300–500 mL if other animal-source foods are not included [13]. A minimum of two milk feeds, including any formula (e.g., infant formula, follow-on formula, “toddler milk”) or any animal milk other than human breast milk (e.g., cow milk, goat milk, evaporated milk, or reconstituted powdered milk), as well as semi-solid and fluid/drinkable yogurt and other fluid/drinkable fermented products made with animal milk, would generally be necessary to provide 200–500 mL per day [15]. However, there is no study conducted in sub-Saharan Africa (SSA) to determine minimum milk feeding frequency (MMFF) and its determinants using the most recent WHO and UNICEF guidelines for assessing infant and young child feeding practices as far as our search is concerned. Therefore, this study is intended to determine the magnitude and associated factors of minimum milk feeding frequency among non-breastfed children aged 6–23 months in SSA using the most recent guideline and Demographic and Health Surveys (DHS) dataset.

## Methods and materials

### Data sources, study design, and sampling

A cross-sectional pooled dataset using the most recent DHS from 20 SSA countries, which was conducted between 2015 and 2022, was used. Demographic and health surveys data from 20 SSA countries, including Angola (2015-16), Benin (2017-18), Burundi (2016-17), Ethiopia (2016), Gabon (2019-21), Gambia (2019-20), Guinea (2018), Kenya (2022), Liberia (2019-20), Mali (2018), Malawi (2015-16), Nigeria (2018), Rwanda (2019-20), Sierra Leone (2019), Senegal (2019), Tanzania (2022), Uganda (2016), South Africa (2016), Zambia (2018), and Zimbabwe (2015), were used. The data were appended to figure out the pooled prevalence of minimum milk feeding frequency and its associated factors in SSA countries. Different datasets, including those for children, males, women, births, and households, are included in the survey for each country. For this study, the kid’s record (KR) file was used. The DHS is a nationwide survey, mostly collected every five years across low- and middle-income countries. It makes cross-country comparison possible as it uses standard procedures for sampling, questionnaires, data collection, cleaning, coding, and analysis [16].

A total weighted sample of 13,315 non-breastfed children aged 6–23 months was included in the study (Table 1). The DHS employs a stratified, two-stage sampling technique [17]. The first stage involves the development of a sampling frame, consisting of a list of primary sampling units (PSUs) or enumeration areas (EAs), which covers the entire country and is usually developed from the latest available national census. The second stage is the systematic sampling of households listed in each cluster, or EA. Further information on the survey sampling strategies is available in the DHS guideline [18].

**Table 1:** Sample size for minimum milk feeding frequency and its associated factors among non-breastfed children aged 6 to 23 months in sub-saharan African countries

Country	Year of survey	Weighted sample (*n*)	Weighted sample (%)
Angola	2015-16	942	7.07
Benin	2017-18	829	6.23
Burundi	2016-17	306	2.30
Ethiopia	2016	364	2.73
Gabon	2019-21	1,140	8.56
Gambia	2019-20	377	2.83
Guinea	2018	345	2.59
Kenya	2022	477	3.58
Liberia	2019-20	308	2.31
Mali	2018	461	3.47
Malawi	2015-16	628	4.72
Nigeria	2018	2,453	18.42
Rwanda	2019-20	175	1.32
Sierra Leone	2019	665	4.99
Senegal	2019	319	2.40
Tanzania	2022	798	5.99
Uganda	2016	937	7.04
South Africa	2016	515	3.87
Zambia	2018	734	5.52
Zimbabwe	2015	543	4.08
Total sample size		13,315	100.00

### Variables of the study

#### Dependent variable

the outcome variable was minimum milk feeding frequency, which is the percentage of non-breastfed children aged 6 to 23 months of age who consumed at least two milk feeds during the previous day [15]. It was measured based on the maternal report and coded as 1 “if the child consumed at least two milk feeds during the previous day” and 0 “if otherwise.” Milk feeds include any formula (e.g., infant formula, follow-on formula, “toddler milk”) or any animal milk other than human breast milk (e.g., cow milk, goat milk, evaporated milk, or reconstituted powdered milk), as well as semi-solid and fluid/drinkable yogurt and other fluid/drinkable fermented products made with animal milk. The milk-related foods available in the DHS are tinned, powdered, or fresh milk; baby formula; cheese, yogurt, and other milk products; and yogurt.

#### Independent variables

both individual and community-level variables were considered in this study. Individual-level factors: maternal age (< 20 years, 20–34 years, 34 + years), maternal education (no formal education, primary, secondary+), maternal working status (not working, working), marital status (unmarried, married), household wealth status (poor, middle, rich), media exposure (no, yes), pregnancy intention (unintended, intended), ANC visit (no visit, 1 to 3 visits, 4 and above visits), PNC checkup (no, yes), place of delivery (home, health facility), age of the child (6–8 months, 9–11 months, 12–17 months, 18–23 months), preceding birth interval (≤ 24 months, > 24 months), and sex of the child (male, female). Community-level factors: place of residence (urban, rural), community-level media exposure (low, high), community-level education (low, high), and community poverty level (low, high). These factors were created by aggregating individual-level factors, as these factors were not directly accessible from DHS data.

### Operational definitions

#### Non-breastfed children

Living children living with their mother, born 6 to 23 months before the survey, who are not breastfeeding.

#### Household wealth status

It is categorized as poor (poorest and poorer), middle, and rich (richer and richest) wealth status.

#### Media exposure

Generated by combining whether a respondent reads newspapers or magazines, listens to the radio, or watches television, and is coded as “yes” if the mother was exposed to at least one of these media and “no” otherwise.

#### Pregnancy intention

Re-categorized as intended (if the pregnancy was wanted) and unintended (incorporating both mistimed and unintended).

#### Community-level of media exposure

The proportion of women who had been exposed to at least one media (television, radio, or newspaper) and categorized based on the national median value as low (communities with ≤ 50% of women exposed) and high (communities with > 50% of women exposed).

#### Community-level education

The proportion of women with a minimum primary level of education derived from data on respondents’ level of education. Then, it was categorized using the national median value into two categories: low (communities with ≤ 50% of women having at least primary education) and high (communities with > 50% of women having at least primary education).

#### Community poverty level

An aggregated variable from household wealth status (proportion of women from poor and rich wealth status), and it was recoded as low and high community poverty level, likewise.

### Data management and analysis

Data extracted from the most recent DHS data sets were cleaned, recoded, and analyzed using STATA/SE version 14.0 statistical software. Sample weight was employed to manage sampling errors and non-responses. Continuous variables were categorized, and categorical variables were further re-categorized. Descriptive analysis was carried out to present the data in frequencies and percentages. Both the individual and community-level variables were presented using descriptive statistics. The DHS data’s variables were organized in clusters; 13,315 children are nested within households, and households were nested within 1691 clusters. The assumptions of independent observations and equal variance across clusters were broken to employ the traditional logistic regression model. This is an indication that using a sophisticated model to take into account between-cluster factors is necessary. As a result, multilevel mixed-effects logistic regression was used to determine the factors associated with MMFF. Multilevel mixed effect logistic regression follows four models: the null model (outcome variable only), model I (only individual-level variables), model II (only community-level variables), and model III (both individual and community-level variables). The model without independent variables (the null model) was used to check the variability of MMFF across the cluster. The association of individual-level variables with the outcome variable (Model I) and the association of community-level variables with the outcome variable (Model II) were assessed. In the final model (Model III), the association of both individual and community-level variables was fitted simultaneously with the outcome variable (MMFF).

The magnitude of the clustering effect and the degree to which community-level factors explain the unexplained variance of the null model were quantified by checking the intra-class correlation coefficient (ICC) and proportional change in variance (PCV). A model with the lowest deviance was selected as the best-fitted model. Finally, variables with a p-value less than 0.05 and an adjusted odds ratio (AOR) with a 95% confidence interval (CI) were described as statistically significant variables associated with MMFF. The presence of multi-collinearity between covariates was checked by using a variance inflation factor (VIF) falling within acceptable limits of 1–10, indicating the absence of significant collinearity across independent variables.

### Random-effect results

Random effects or measures of variation of the outcome variable were estimated using the median odds ratio (MOR), ICC, and PCV. The variation between clusters was measured by the ICC and PCV. Taking clusters as a random variable, the ICC reveals that the variation of minimum milk feeding frequency between clusters is computed as ICC = VC/(VC + 3.29) ×100%. The MOR is the median value of the odds ratio between the area of the highest risk and the area of the lowest risk for MMFF when two clusters are randomly selected, using clusters as a random variable; MOR = 𝑒 0.95√VC. In addition, the PCV demonstrates the variation in the prevalence of MMFF explained by factors and is computed as: PCV = (Vnull-VC)/Vnull×100%; where Vnull = variance of the null model and VC = cluster level variance [19]. The fixed effects were used to estimate the association between the likelihood of MMFF and individual and community-level independent variables.

## Results

### Individual- and community-level characteristics of study subjects

A total of 13,315 non-breastfed children aged 6 to 23 months were included in this study. The mean age of mothers was 28.84 ± 0.05 years, and 74.08% of them fall in the age range of 20–34 years. Nearly half (47.11%) of the mothers had completed secondary or higher education, and 82.12% of them were currently married. More than two-thirds (69.12%) of the mothers had jobs, and 71.69% of them had media exposure. More than one-third (35.48%) of the mothers had poor wealth status, and 67.56% of them had intended pregnancy. Health facility delivery was reported by 76.20% of mothers in SSA countries, and 66.49% of them attended 4 + ANC visits during their last pregnancy. Only 38.23% of them had a PNC checkup after birth. The mean age of children was 17.78 ± 0.04 months, and 64.14% of them fall in the age range of 18–23 months. More than half (51.44%) of the children were males, and the preceding birth interval was > 24 months for the majority (84.02%) of non-breastfed children aged 6 to 23 months. More than half (52.14%) of the study subjects were from rural areas, and 50.17% of them were from a community with low media exposure. More than half (50.68%) and 52.49% of mothers of non-breastfed children aged 6 to 23 months had low community-level education and low community poverty, respectively (Table 2).

**Table 2:** Individual-and community-level characteristics of study subjects, pooled data from 20 SSA countries, DHS 2015–2022

Variables	Category	Frequency (*n*)	Percentage (%)
Maternal age	< 20 years	933	7.01
	20–34 years	9,864	74.08
	34 + years	2,518	18.91
Maternal educational level	No formal education	3,317	24.91
	Primary	3,725	27.98
	Secondary+	6,273	47.11
Marital status of the mother	Unmarried	2,382	17.89
	Married	10,933	82.12
Maternal occupation	Not working	4,112	30.88
	Working	9,203	69.12
Exposure to media	Yes	9,545	71.69
	No	3,770	28.31
Wealth index	Poor	4,725	35.48
	Middle	2,527	18.98
	Rich	6,064	45.54
Pregnancy intention	Unintended	4,320	32.44
	Intended	8,995	67.56
Place of delivery	Home	3,169	23.80
	Health facility	10,146	76.20
ANC visits	No visit	1,477	11.10
	1 to 3 visits	2,984	22.41
	4 and above visits	8,854	66.49
PNC checkup	Yes	4,905	38.23
	No	7,926	61.77
Current Age of the child	6–8 months	549	4.12
	9–11 months	675	5.07
	12–17 months	3,551	26.67
	18–23 months	8,540	64.14
Sex of the child	Male	6,850	51.44
	Female	6,465	48.56
Preceding birth interval	≤ 24 months	2,128	15.98
	> 24 months	11,187	84.02
Place of residence	Urban	6,372	47.86
	Rural	6,943	52.14
Community media exposure	Low	6,680	50.17
	High	6,635	49.83
Community level education	Low	6,748	50.68
	High	6,567	49.32
Community poverty	Low	6,988	52.49
	High	6,327	47.51

### Pooled magnitude of minimum milk feeding frequency

The overall pooled estimate of the minimum milk feeding frequency among non-breastfed children aged 6–23 months in sub-Saharan African countries was 12.39% (95% CI: 11.85%, 12.97%) and ranged from 2.08% in Tanzania to 44.60% in South Africa.

### Measures of variation and model fitness

A null model was used to determine whether the data supported the decision to assess randomness at the community level. Findings from the null model showed that there were significant differences in minimum milk feeding frequency between communities, with a variance of 0.2352656 and a P value of < 0.001. The variance within clusters contributed to 93.33% of the variation in minimum milk feeding frequency, while the variance across clusters was responsible for 6.67% of the variation. In the null model, the odds of minimum milk feeding frequency differed between higher and lower-risk clusters by a factor of 1.58. The intra-class correlation value for Model I indicated that 5.16% of the variation in minimum milk feeding frequency accounts for the disparities between communities. Then, with the null model, community-level variables were used to generate Model II. According to the ICC value from Model II, cluster variations were the basis for 6.06% of the differences in minimum milk feeding frequency. In the final model (model III), which attributed approximately 5.00% of the variation in the likelihood of minimum milk feeding frequency to both individual and community-level variables, the likelihood of minimum milk feeding frequency varied by 1.48 times across low and high minimum milk feeding frequencies (Table 3).

**Table 3:** Model comparison and random effect analysis for minimum milk feeding frequency and its associated factors in sub-saharan African countries, DHS 2015–2022 (*n* = 13,315)

Parameter	Null model	Model I	Model II	Model III
Variance	0.2352656	0.1791282	0.2122501	0.1732301
ICC	6.67%	5.16%	6.06%	5.00%
MOR	1.58	1.49	1.55	1.48
PCV	Reference	23.86%	9.78%	26.37%
Model fitness				
LLR	-4980.7588	-4377.4217	-4725.9094	-4312.2512
Deviance	9,961.5176	8,754.8434	9,451.8188	8,624.5024

ICC: Intra cluster correlation, LLR: log-likelihood ratio, MOR: median odds ratio, PCV: Proportional change in variance

### Individual and community-level factors associated with minimum milk feeding frequency

In the final fitted model of multivariable multilevel logistic regression, maternal educational level, current marital status of the mother, maternal working status, media exposure, wealth index, place of delivery, ANC visits attended during pregnancy, PNC checkup, the current age of the child, and residence were significantly associated with minimum milk feeding frequency among non-breastfed children aged 6–23 months. Accordingly, the odds of minimum milk feeding frequency were 1.61 times higher among mothers with secondary and higher education compared with mothers who had no formal education [AOR = 1.61; 95% CI (1.35, 1.91)]. Married women were 23% less likely to meet the minimum milk feeding frequency than their counterparts [AOR = 0.77; 95% CI (0.67, 0.89)]. Mothers who had jobs were 20% less likely to meet the minimum milk feeding frequency than those who hadn’t jobs [AOR = 0.80; 95% CI (0.71, 0.91)].

Children of mothers with media exposure (radio, television, newspaper) were 1.50 times more likely to meet the minimum milk feeding frequency compared with their counterparts [AOR = 1.50; 95% CI (1.27, 1.77)]. Children from families with rich wealth status were 1.21 times more likely to access the minimum milk feeding frequency compared with those from families with poor wealth status [AOR = 1.21; 95% CI (1.03, 1.42)]. Children of mothers who gave birth at a health facility were 1.45 times more likely to meet the minimum milk feeding frequency than those who gave birth at home [AOR = 1.45; 95% CI (1.22, 1.72)]. Children of mothers who attended 1 to 3 and 4 + ANC visits during pregnancy were 51% and 33% times less likely to access minimum milk feeding frequency than those who had no ANC visit, respectively [AOR = 0.49; 95% CI (0.39, 0.62)] and [AOR = 0.67; 95% CI (0.55, 0.82)]. A postnatal care checkup increases the odds of minimum milk feeding frequency by 1.57 times [AOR = 1.57; 95% CI (1.40, 1.76)]. Children aged 18–23 months were 30% less likely to fulfill the minimum milk feeding frequency than those aged 6–8 months [AOR = 0.70; 95% CI (0.53, 0.93)]. Children who lived in urban residences were 2.15 times more likely to access minimum milk feeding frequency compared with those from rural areas [AOR = 2.15; 95% CI (1.87, 2.46)] (Table 4).

**Table 4:** Multivariable multilevel logistic regression analysis of individual and community-level factors associated with minimum milk feeding frequency among non-breastfed children aged 6 to 23 months in SSA countries, DHS 2015–2022 (*n* = 13,315)

Variables	Category	Model I AOR (95% CI)	Model II AOR (95% CI)	Model III AOR (95% CI)
Maternal age	< 20 years	1		1
	20–34 years	1.05 (0.84, 1.32)		1.04 (0.83, 1.31)
	34 + years	1.09 (0.84, 1.41)		1.07 (0.83, 1.39)
Maternal educational level	No formal education	1		1
	Primary	0.59 (0.48, 0.72)*		0.61 (0.50, 0.75)*
	Secondary+	1.66 (1.40, 1.96)*		1.61 (1.35, 1.91)*
Marital status of the mother	Married	0.73 (0.63, 0.84)*		0.77 (0.67, 0.89)*
	Unmarried	1		1
Maternal occupation	Not working	1		1
	Working	0.76 (0.67, 0.86)*		0.80 (0.71, 0.91)*
Exposure to media	Yes	1.64 (1.40, 1.93)*		1.50 (1.27, 1.77)*
	No	1		1
Wealth index	Poor	1		1
	Middle	1.07 (0.90, 1.27)		0.97 (0.81, 1.16)
	Rich	1.65 (1.43, 1.90)*		1.21 (1.03, 1.42)*
Pregnancy intention	Unintended	1		1
	Intended	1.09 (0.96, 1.23)		1.08 (0.95, 1.22)
Place of delivery	Home	1		1
	Health facility	1.53 (1.29, 1.81)*		1.45 (1.22, 1.72)*
ANC visits	No visit	1		1
	1 to 3 visits	0.48 (0.38, 0.60)*		0.49 (0.39, 0.62)*
	4 and above visits	0.67 (0.55, 0.82)*		0.67 (0.55, 0.82)*
PNC checkup	No	1		1
	Yes	1.61 (1.44, 1.81)*		1.57 (1.40, 1.76)*
Age of child	6–8 months	1		1
	9–11 months	1.39 (0.99, 1.96)		1.37 (0.97, 1.92)
	12–17 months	0.98 (0.73, 1.30)		0.95 (0.71, 1.26)
	18–23 months	0.70 (0.53, 0.93)*		0.70 (0.53, 0.93)*
Sex of child	Male	0.96 (0.86, 1.07)		0.97 (0.87, 1.08)
	Female	1		1
Preceding birth interval	≤ 24 months	1		1
	> 24 months	1.06 (0.90, 1.25)		1.08 (0.91, 1.27)
Place of residence	Urban		3.51 (3.12, 3.94)*	2.15 (1.87, 2.46)*
	Rural		1	1
Community media exposure	Low		1	1
	High		1.15 (1.00, 1.33)*	1.03 (0.89, 1.19)
Community level education	Low		1	1
	High		0.94 (0.81, 1.07)	0.88 (0.77, 1.02)
Community poverty	Low		0.95 (0.83, 1.09)	0.91 (0.79, 1.05)
	High		1	1

ANC: Antenatal care; PNC: Postnatal care; *statistically significant at p-value < 0.05

## Discussion

To our knowledge, this is the first study conducted in SSA on minimum milk feeding frequency in accordance with the most recent WHO and UNICEF guidelines for assessing infant and young child feeding practices, which were published in 2021 [15]. In the current study, the pooled magnitude of minimum milk feeding frequency among non-breastfed children aged 6 to 23 months in SSA countries was 12.39% (95% CI: 11.85%, 12.97%). This finding was lower than a study conducted in Nepal (17.8%) [20]. The possible justification for this discrepancy might be differences in sample size, geographical differences, population growth, and sociocultural differences. Thus, there is a necessity for behavioral modification communication involvement, especially in rural areas of SSA, like mass media campaigns among communities, particularly among reproductive-age women.

The present study also identified individual and community-level factors associated with MMFF. The odds of MMFF were higher among mothers with a higher educational level. This finding was consistent with a study conducted in Nepal [20]. This might be due to the fact that women with higher educational status are more likely to have access to better healthcare services and communications, as they can easily understand and convert that evidence into practice [21]. Besides, the advancement of ideal child nutrition and consequent uptake of nutrition information, as well as the health-seeking behavior of mothers with higher educational status, can contribute to the higher odds of MMFF [22]. Married women were less likely to meet the MMFF than their counterparts. This finding contradicts the findings of a study conducted in SSA [23]. This finding can be explained by the higher levels of women’s decision-making authority are associated with better nutritional outcomes. Married women may have less decision-making power in their home and be less likely to meet MMFF for their child [24]. Working mothers were less likely to meet the minimum milk feeding frequency. This finding was consistent with a study conducted in the Philippines [25]. This may be because mothers who do not work have a greater propensity to stay at home with their children, providing them with a healthy diet, which may help them reach their MMFF. Children of mothers with media exposure were more likely to meet MMFF compared with their counterparts. Studies conducted in the SSA [23] and Nepal [20] reported similar findings. This might be attributed to the reliable nutrition-related information adopted by mothers from the media (radio, television, newspapers, or magazines) [21]. Mothers who are exposed to the media may learn things that alter their behavior and enable them to give their children MMFF. Children from families with rich wealth status were more likely to access MMFF compared with those from families with poor wealth status. This finding was similar to studies conducted in Ethiopia [26] and SSA [23]. This might be due to the fact that meeting dietary requirements depends on the economic status of households. Mothers with rich wealth status can feed their children varied foods more frequently, as they are more likely to have enough money to buy diversified foods as compared to mothers with poor wealth status [27]. Institutional delivery also increased the odds of MMFF. This might be due to healthcare professionals counseling about child health and nutrition during health facility delivery, which advances infant and young children’s feeding practices, including meeting the MMFF for non-breastfed children.

Children of mothers who attended ANC visits during pregnancy were less likely to access MMFF. This finding contradicts a study conducted in SSA [23]. This implies that the focus should be on the quality of ANC rather than counting the number of ANC visits attended by pregnant women. The quality of maternal nutrition and infant feeding counseling provided during antenatal care is limited by disparities in service accessibility, a lack of chances for frontline workers to develop their abilities, and the short-term and irregular nature of counseling contracts [28]. Indicators for monitoring and supervision systems, recurring surveys, and program evaluations require more focus in order to track the status of quality improvement, establish accountability for high-quality counseling, and assess its current state. Postnatal care checkups also had an association with MMFF, which increased the odds of minimum milk feeding frequency by 1.57 times. A study conducted in Ethiopia [26] reported a similar finding. This can be explained by nutrition counseling regarding infant and child feeding practices, as it is one of the components of a PNC checkup. Mothers who had a PNC checkup had a higher chance of obtaining nutritional advice on infant and young child feeding practices, which encouraged them to give the daily recommended MMFF for their children. Children aged 18–23 months were less likely to access MMFF than children aged 6–8 months. This finding was consistent with a study conducted in Germany, in which diary intake decreased with age [29]. Evidence shows that a decrease in dairy intake appears to have health benefits without clearly stating how much dairy should be consumed as the child gets older [30]. Finally, children of mothers who lived in urban residences were more likely to access MMFF compared with those from rural areas. This finding was in agreement with studies conducted in Ethiopia [26] and SSA [23]. This might be described by the better socioeconomic status, nutrition information access, and educational attainment of mothers who live in urban areas than their counterparts, which in turn results in better caring practices for their children. Furthermore, mothers who live in urban areas have a better chance to get nutrition-related information from different reliable information sources like health care professionals, the media, and the internet.

### Strengths and limitations of the study

Firstly, this study is representative at sub-Saharan and regional levels as it uses weighted nationally representative data for each SSA country with a large sample. Secondly, this study can be generalizable in the study setting to all children aged 6 to 23 months during the study period, as the study has appropriate statistical power. Thirdly, the findings of this study will provide vital information for region-specific intervention since the pooled estimate of minimum milk feeding frequency in SSA is reported. The study also has some limitations. There might be a possibility of recall and social desirability bias since the data were self-reported. The secondary nature of the data was also another limitation of this study. Besides, the cross-sectional nature of the study couldn’t enable us to establish the cause-and-effect relationship between the outcome variable and independent variables. Finally, some important variables that affect the outcome variable, like religion and fasting, are missed.

## Conclusions

In sub-Saharan Africa, the proportion of minimum milk feeding frequency among non-breastfed children aged between 6 and 23 months was low. The likelihood of minimum milk feeding frequency increases with high levels of education, unemployment, media exposure, rich wealth status, being unmarried, having a child born in a health facility, getting PNC checks, being between 6 and 8 months old, and living in an urban area. Hence, promoting women’s education, increasing economic status of the household, disseminating nutrition information through media, strengthening maternal health service utilization like health facility delivery and PNC services, and giving prior attention to mothers with older children and from rural areas are recommended to increase the magnitude of the minimum milk feeding frequency among non-breastfed children aged 6–23 months in SSA. Further research is needed to explore the barriers to minimum milk feeding frequency and focus on interventions to improve the feeding practices of non-breastfed children among sub-Saharan African mothers.

### Electronic supplementary material

Below is the link to the electronic supplementary material.

Supplementary Material 1

## Data Availability

The data from the 20 SSA countries is publicly available online at https://dhsprogram.com/data/available-datasets.cfm.

## Abbreviations

ANC: Antenatal Care
AOR: Adjusted Odds Ratio
CI: Confidence Interval
DHS: Demographic and Health Survey
ICC: Intra-class Correlation Coefficient
MMFF: Minimum Milk Feeding Frequency
MOR: Median Odds Ratio
PCV: Proportional Change in Variance
PNC: Postnatal Care
SSA: Sub-Saharan Africa
VIF: Variance Inflation Factor
WHO: World Health Organization
