# Supplementary material for: Minimum milk feeding frequency and its associated factors among non-breastfed children aged 6–23 months in sub-saharan Africa: a multilevel analysis of the recent demographic and health survey data
Source: BMC Public Health. 2024 Jun 28;24:1734. doi: 10.1186/s12889-024-19275-2 (PMC11214211; doi:10.1186/s12889-024-19275-2)
Supplement: Supplementary file 1 — Supplementary Material 1 [file 12889_2024_19275_MOESM1_ESM.docx]

**Do files**

**Null model:** melogit MMFF ||v001:,or

**Model I**: melogit MMFF i.maternal_age i.maternal_education i.maternal_occupation i.marital_status i.wealth_index i.media_exposure i.pregnancy_intention i.ANC_visits i.PNC_checkup i.place_delivery i.age_child i.birth_interval ib2.sex_child ||v001:,or

**Model II**: melogit MMFF ib2.residence i.community_ME i.community_education ib1.community_poverty ||v001:,or

**Model III**: melogit MMFF i.maternal_age i.maternal_education i.maternal_occupation i.marital_status i.wealth_index i.media_exposure i.pregnancy_intention i.ANC_visits i.PNC_checkup i.place_delivery i.age_child i.birth_interval ib2.sex_child ib2.residence i.community_ME i.community_education ib1.community_poverty ||v001:,or
